# Renal Protective Effects of Melatonin in Animal Models of Diabetes Mellitus-Related Kidney Damage: A Systematic Review and Meta-Analysis

**DOI:** 10.1155/2022/3770417

**Published:** 2022-06-14

**Authors:** Qian Luo, Yuzi Cai, Qihan Zhao, Yuhua Jiang, Lei Tian, Yuning Liu, Wei Jing Liu

**Affiliations:** ^1^Dongzhimen Hospital Affiliated to Beijing University of Chinese Medicine, Beijing University of Chinese Medicine, Beijing 100700, China; ^2^Key Laboratory of Chinese Internal Medicine of Ministry of Education and Beijing, Beijing 100700, China; ^3^Zhanjiang Key Laboratory of Prevention and Management of Chronic Kidney Disease, Guangdong Medical University, Zhanjiang, Guangdong 524001, China

## Abstract

Diabetic nephropathy (DN)—chronic kidney damage caused by hyperglycemia—eventually develops into end-stage renal disease (ESRD). Melatonin is a powerful antioxidant that has a wide range of biological activities. Potentially helpful effects of melatonin on diabetic kidney disease have been found in several studies. However, its protective mechanisms are not clear and remain to be explored. In this review (CRD42021285429), we conducted a meta-analysis to estimate the effects and relevant mechanisms of melatonin for diminishing renal injuries in diabetes mellitus models. The Cochrane Library, PubMed, and EMBASE databases up to September 2021 were used. Random- or fixed-effects models were used for calculating the standardized mean difference (SMD) or 90% confidence interval (CI). The risk of bias was estimated using the SYRCLE's RoB tool. Statistical analysis was conducted with RevMan. A total of 15 studies including 224 animals were included in the analysis. The experimental group showed a remarkable decrease in serum creatinine (*P* = 0.002), blood urea nitrogen (*P* = 0.02), and urinary albumin excretion rate (UAER) (*P* < 0.00001) compared with the control group, while the oxidative stress index improved. The experimental group also showed a remarkable increase in superoxide dismutase (*P* = 0.21), glutathione (*P* < 0.0001), and catalase (*P* = 0.04) and a remarkable decrease in MDA (*P* < 0.00001) content compared with the control group. We concluded that melatonin plays a role in renal protection in diabetic animals by inhibiting oxidative stress. Moreover, it should be noted that fasting blood glucose was reduced in the experimental group compared with the control group. The kidney and body weights of the animals were not decreased in the diabetic animal model compared with the control group.

## 1. Introduction

Diabetes mellitus (DM) is a metabolic disorder characterized by hyperglycemia that is caused by insulin resistance or declined insulin secretion. The International Diabetes Mellitus Federation predicts that 592 million people will suffer from DM by 2035 [1–4]. Diabetic nephropathy, a common complication of DM, is one of the most serious causes of chronic kidney disease (CKD) and end-stage kidney disease (ESKD) in the world [5, 6]. At present, the treatment strategy for diabetic nephropathy is mainly based on strict control of blood glucose and blood pressure [7], but its curative effect is still controversial. Therefore, it is of clinical significance to explore the process and mechanism of kidney injury induced by high glucose levels. Some basic mechanisms, such as the accumulation of advanced glycation end products (AGEs), the abnormal production of specific growth factors/cytokines, and the complex hemodynamic or disordered endocrine system, have been recognized, and they may lead to a vicious cycle that includes oxidative stress, persistent inflammatory reaction, and incompetent apoptosis, finally leading to persistent proteinuria, decreasing estimated glomerular filtration rate, high blood pressure, and an abnormal blood lipid level [8–10]. It is worth noting that increased oxidative stress in DM has been shown to play a critical role in the pathogenesis of diabetic kidney disease and has recently been considered a promoting factor for the progression of diabetic kidney disease [11]. The antioxidant system of patients with diabetes has defects, and hyperglycemia can result in the increase of free radicals, which can further lead to lipid peroxidation and promotion of oxidative stress [11–13]. Kedziora et al. found that the activity of the kidney antioxidant system of streptozotocin-induced diabetic rats decreased and lipid peroxidation increased [14, 15]. In recent years, the effects of antioxidants against oxidative stress in diabetic nephropathy have been reported in many experimental models. For instance, *Ginkgo biloba* and garlic extracts can improve glomerular hypertrophy in a diabetic rat model [16, 17], and taurine and vitamin E can effectively reduce collagen production in rat mesangial cells induced by high glucose [18, 19]. These reports illustrate the necessity and feasibility of antioxidant research for treating diabetic nephropathy.

Melatonin is a hormone produced by the pineal gland and some other organs [20]. It has a strong antioxidant effect and reduces lipid peroxidation [14]. There is increasing evidence showing that the antioxidant activity of melatonin may be beneficial for treating diabetes and losing weight [21, 22]. According to Katarzyna et al., melatonin could contribute to the treatment of diabetes by regulating glucose metabolism and antioxidative stress [7], and it has a role in kidney protection, which can alleviate symptoms in the early stage of glomerular diseases [23]. However, the available data have not been systematically analyzed. Therefore, helpful information for later clinical studies on the use of melatonin (as a nutritive or supplement) could be provided by performing a meta-analysis of animal studies. This review is aimed at comprehensively exploring the role of melatonin in animal models of diabetic nephropathy.

## 2. Methods

### 2.1. Data Sources and Search

The databases, such as Cochrane Library, PubMed, and EMBASE, were searched for studies of the use of melatonin in animal models of renal damage in diabetes mellitus up to September 2021.

### 2.2. Study Selection

The inclusion criteria were as follows: (1) the research model was diabetic animal model (DAM); (2) DAM could be established by diverse methods; (3) the treatment group received melatonin alone, while the control group was given saline or no treatment; and (4) the primary outcomes were serum creatinine (Scr), blood urea nitrogen (BUN), and UAER, and the secondary outcomes were fasting blood glucose (FBG), kidney weight, body weight, and oxidative stress indexes. The exclusion criteria were as follows: (1) other types of studies (e.g., cases, reviews, cell studies, and clinical trials); (2) other disease models; (3) other treatment drugs; and (4) no relevant outcomes reported.

### 2.3. Data Extraction

Two authors extracted the following details: (1) study characteristics (first author, publication year, and sample size); (2) basic characteristics of the included animals; (3) methods of modeling; (4) intervention (such as route and dosage), and (5) primary and secondary outcomes. All data for which outcomes were acquired under the intervention of different dosage subgroups were extracted. If the data were given in a graph, authors were contacted for information. Data were extracted by using digital ruler software if authors were not contacted. The treatment was carried out by the time of successful establishment of the model. Disputes arising in the process of data extraction were resolved by negotiation or by a third person.

### 2.4. The Risk of Bias Assessment

**Figure 1:**
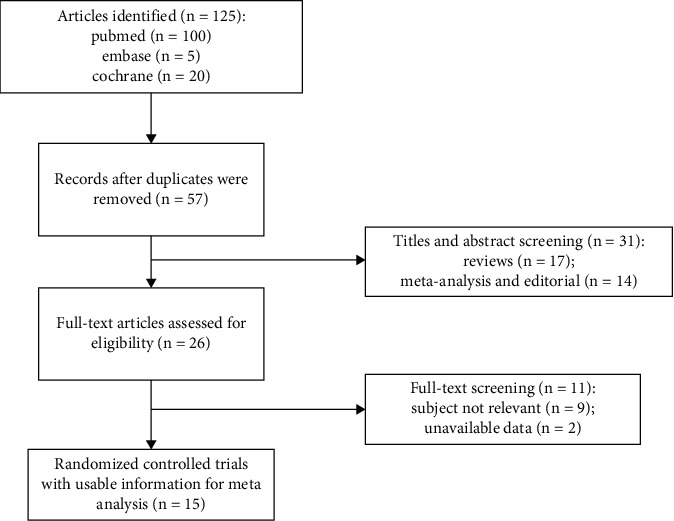
Risk-of-bias summary using the SYRCLE risk of bias tool.

Two authors (Cai and Luo) separately evaluated the risk of bias of the included studies using the SYRCLE's risk of bias tool [24] according to 10 domains (Figure 1); the risks were classified as either “low,” “high,” or “unclear.” Zhao and Tian resolved any discrepancies if there was disagreement between Luo and Cai.

### 2.5. Subgroup and Sensitivity Analysis

Due to the limited number of the included studies, the single sex of experimental animals (male), incomplete information on age and weight, and inconsistent routes of melatonin intake, it was difficult to carry out subgroup analysis in this review. Where there was marked heterogeneity in the main results (*I*^2^ > 50%), sensitivity analysis was conducted and the stability of the results by omitting each study in sequence was evaluated.

### 2.6. Data Synthesis

The data entry and analysis were performed using Excel 2016, Stata statistical software version 12.0, and RevMan 5.3. All outcomes were continuous variables. Where the data in the included literature were reported as mean ± SEM, we transformed SEM into SD using the formula (SEM = SD/square root of the sample size) to avoid muddling the distinctive usage between SD and SEM. Statistical heterogeneity was assessed by the chi-square test and the *I*^2^ tests. A fixed-effect model was selected if *I*^2^ was <50%; otherwise, the random-effect model was selected. *P* < 0.05 was considered statistically significant. Several independent groups in a study (e.g., different melatonin doses) were considered separate datasets. This study divided the number of animals in the control group by the number of animals in the comparison groups in each study to effectively solve the artificial increase of sample size in comprehensive analysis.

## 3. Results

**Figure 2:**
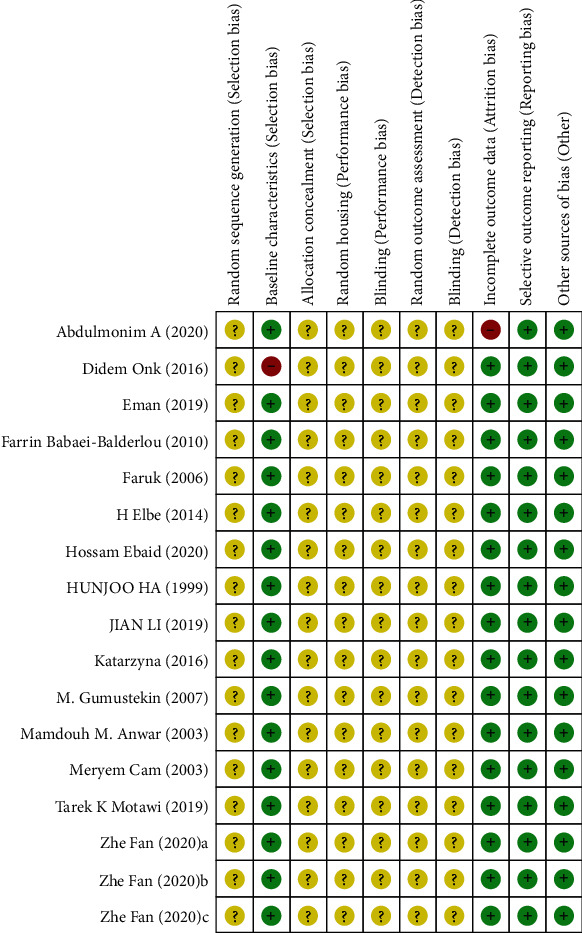
Flowchart of literature search and selection.

A total of 125 articles were found. After removing duplicates, 57 studies were selected for the next step. After screening titles and abstracts of the selected articles, we removed review articles, cell studies, and human studies, and 26 articles were selected for full-text screening. Finally, 15 eligible manuscripts (Figure 1) evaluating renal protective effects of melatonin in DAM were analyzed. The literature search process is shown in Figure 2.  Table 1 presents the basic characteristics of the final 15 studies.

### 3.1. The Risk of Bias in the Included Trials and the Publication Bias

The risk of bias assessment of the articles included in this study is presented in Figure 1. The studies involved in this review contained insufficient information about the experimental details, and as a result, several studies were judged as having “unclear risk of bias.” Random sequence generation, allocation concealment, random housing, blinding (performance bias), blinding (detection bias), random outcome assessment, and blinding of outcome assessment were incompletely described in all of the studies. Most studies [7, 11, 14, 20, 23–34] had a low risk of bias for baseline characteristics, except one [25]. One study [23] had a high risk of bias for incomplete outcome data due to animal death, while other studies had a low risk. In addition, fewer than 10 papers included the main indicators analyzed in our study, so funnel chart analysis was not carried out.

### 3.2. Effectiveness

#### 3.2.1. Primary Outcomes (Figures 3–5)

*(1) Scr*. Analysis of eight studies [7, 20, 23–26, 28, 32, 33] showed that the melatonin group had a markedly reduced Scr level in DAM compared with the control group (*n* = 102; SMD, −2.45; 95% confidence interval (CI), −3.97 to −0.93; *I*^2^ = 85%, *P* = 0.002). Due to the high heterogeneity, we analyzed the Scr subgroups according to different intake methods of melatonin. The results showed that the heterogeneity of the single intraperitoneal injection subgroup and the oral administration subgroup did not differ significantly.

*(2) BUN*. Analysis of seven studies [7, 20, 23, 26, 28, 32, 33] showed that the melatonin group had a markedly reduced BUN in DAM compared with the control group (*n* = 98; SMD, −1.76; 95% CI, −3.28 to −0.25; *I*^2^ = 87%, *P* = 0.02).

*(3) UAER*. Analysis of four studies [28, 31] showed that the melatonin group had a markedly reduced UAER in DAM compared with the control group (*n* = 60; MD, −510.52; 95% CI, −650.88 to 370.15; *I*^2^ = 81%, *P* < 0.00001).

#### 3.2.2. Secondary Outcomes (Figures 6–12)

*(1) Fasting Blood Glucose*. Analysis of 13 studies [7, 14, 20, 23, 26, 27, 29–33] showed that the melatonin group had a markedly reduced FBG in DAM compared with the control group (*n* = 183; SMD, −1.25; 95% CI, −2.03 to −0.47; *I*^2^ = 76%, *P* = 0.002).

*(2) Kidney Weight*. Analysis of six studies [7, 20, 28, 31] showed that the melatonin group had a reduced kidney weight in DAM compared with the control group, but no significant difference was observed (*n* = 84; MD, −0.02; 95% CI, −0.10 to 0.06; *I*^2^ = 86%, *P* = 0.63).

*(3) Body Weight*. Analysis of eight studies [7, 20, 27, 30–32] showed that the melatonin group did not have a reduction in body weight in DAM compared with the control group (*n* = 109; MD, 11.49; 95% CI, 1.19 to 21.78; *I*^2^ = 88%, *P* = 0.03).

*(4) Oxidative Stress Index Changes in Kidneys*. Analysis of five studies [11, 20, 30, 32, 33] showed that the melatonin group had a markedly reduced MDA (malondialdehyde) level in DAM compared with the control group (*n* = 76; SMD, −1.78; 95% CI, −2.35 to −1.22; *I*^2^ = 0, *P* < 0.00001). Analysis of four studies [11, 20, 32, 33] showed that the melatonin group had an increase in superoxide dismutase (SOD) level in DAM compared with the control group, but no significant difference was observed (*n* = 65; SMD, 1.02; 95% CI, –0.58 to 2.62; *I*^2^ = 87%, *P* = 0.21). Analysis of three studies [20, 30–31] showed that the melatonin group had a markedly increased glutathione (GSH) level in DAM compared with the control group (*n* = 48; SMD, 1.34; 95% CI, 0.68 to 1.99; *I*^2^ = 37%, *P* < 0.0001). Analysis of three studies [20, 32, 34] showed that the melatonin group had a remarkably increased catalase (CAT) level in DAM compared with the control group (*n* = 44; SMD, 1.04; 95% CI, 0.04 to 2.04; *I*^2^ = 56%, *P* = 0.04).

## 4. Discussion

This review was the first to assess the effects of melatonin on renal damage in DAM. It included 15 articles (224 samples) and analyzed seven outcomes (including three primary and four secondary outcome indicators). Our results showed that melatonin can markedly improve kidney function in DAM. The potential reasons are likely related to the antioxidant effects of melatonin. Moreover, it should be noted that FBG in the experimental group was reduced compared with that in the control group, but kidney weight and body weight of the animals were not decreased in DAM compared with the control group.

Melatonin is a powerful antioxidant, and its renal protective properties have been widely discussed [7]. In recent years, many studies have found that melatonin can improve diabetic nephropathy and protect kidney function by reducing urine excretion or protecting podocytes [31]. In addition, there is growing evidence that melatonin plays a protective role in diabetes-related renal fibrosis and glomerular apoptosis [28]. Based on this, our study evaluated the effectiveness of melatonin on renal function in animal models of diabetic kidney injury. The results showed that serum creatinine, urea nitrogen, and urinary protein clearance rate significantly decreased in the melatonin treatment group, and melatonin was beneficial for renal function, which is consistent with the results of many other studies [7, 20, 23–26, 28, 32, 33].

To explain some of the changes caused by melatonin in diabetic animals, this study focused on molecular mechanisms related to oxidative stress that may be affected by melatonin. Increased production of free radicals and decreased activity of the antioxidant system in diabetic nephropathy suggest oxidative stress. The increase in the levels of free radicals leads to lipid peroxidation (LPO), and malondialdehyde is the most common marker of lipid peroxidation. The removal of superoxide mainly depends on antioxidant enzymes, such as superoxide dismutase (SOD), glutathione peroxidase (GPx), and catalase (CAT) [35]. Melatonin, a widely studied antioxidant, has been found to significantly reduce the production of ROS and promote the elimination of ROS [35]. Cam et al. found that melatonin reduced lipid peroxidation and inhibited glomerular basement membrane thickening and mesangial matrix expansion [20]. Hebe et al. found that antioxidants such as melatonin prevented oxidative stress by decreasing lipid peroxidation and increasing SOD and CAT activity levels [20]. Obrosova et al. and Kata reported similar results [20]. Therefore, our study evaluated the changes in the oxidative stress index in kidneys in diabetic kidney injury animal models and found that melatonin reduced the content of MDA and increased GSH level and SOD and CAT activity. Furthermore, the melatonin against receptor of diabetic nephropathy should be considered since the aim of most medical studies is to produce drug and test its efficacy. The actions of melatonin are mediated via two G-protein–coupled membrane receptors, MT1 and MT2 [36], which have been studied well in sleep and circadian rhythms, learning and memory, cancer, depressive disorders, and neuroprotection. Our study did not include MT1/MT2 because there were studies showing that its antioxidant effects are receptor-independent in diabetic nephropathy models [37]. However, the roles of MT1/MT2 receptors in the antioxidant effects of melatonin in a model of diabetic nephropathy should be examined in the future, as several studies of other diseases have found that there is a close association between MT1/MT2 and oxidative stress. For example, Younis et al. [38] found that melatonin and insulin treatment restored the receptor expression pattern of MT1 and MT2, playing an antioxidant effect and reducing liver damage in diabetic rats. Furthermore, Wang et al. [39] found that ramelteon, a melatonin MT1/MT2 receptor agonist, provided cerebral protection after traumatic brain injury by mitigating oxidative stress. In addition to the mechanisms mentioned above, melatonin also has the functions of antiaging, anti-inflammatory, and antihypertensive agents [40, 41]. It has been shown that melatonin can reduce IL-*β*, IL-6, and IL-33, eliminate inflammatory reaction, and inhibit apoptosis by reducing the expression of Bax and caspase-3 and the activity of JAK/STAT. However, there was no further research on this mechanism in this study because it was difficult to acquire a sufficient amount of data.

We also found the effect of antioxidants on lowering blood glucose, but did not find any effects on reducing body weight and kidney weight. Hebe et al. reported similar results [20], which may be related to the short treatment duration and the small sample size.

**Figure 13:**
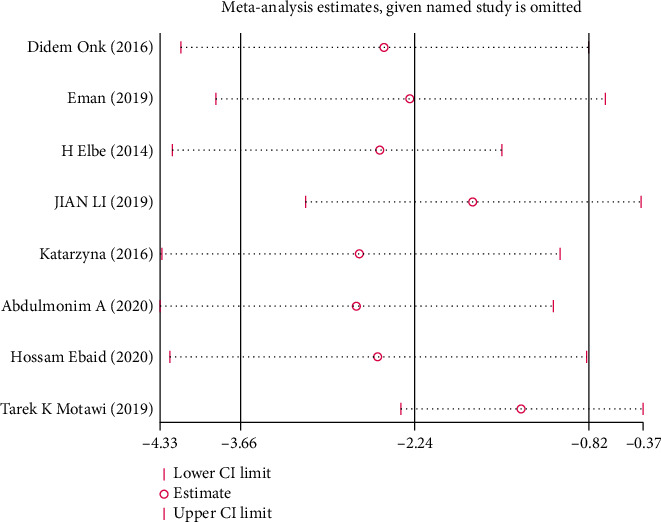
Sensitivity analysis for Scr.

**Figure 14:**
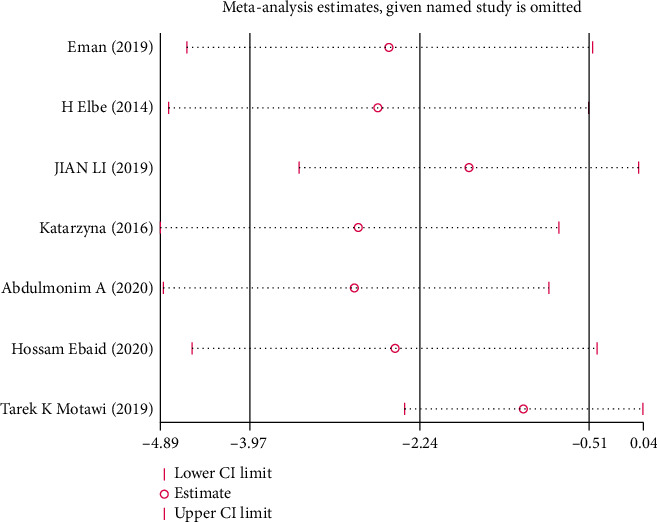
Sensitivity analysis for BUN.

**Figure 15:**
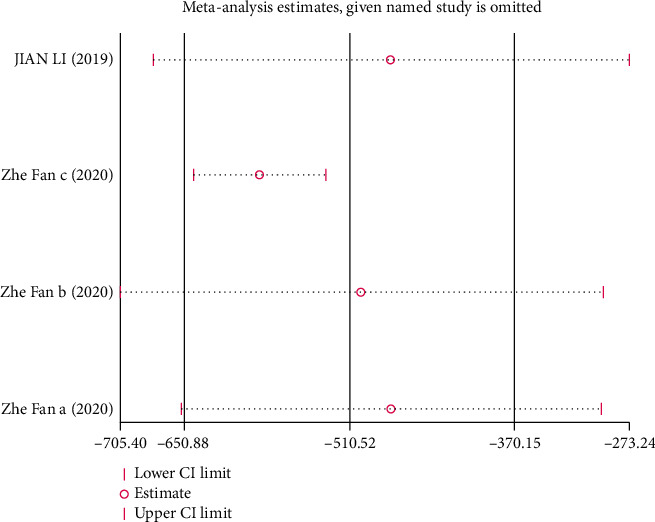
Sensitivity analysis for UAER.

This systematic review has the following limitations: (1) our search only included English and Chinese publications, which may have resulted in language bias; (2) the methodology of the included studies was of low quality, so the reliability of our risk of bias assessment was limited; (3) considering the small total sample size and few studies on mice, we could not investigate whether there were marked differences in the efficacy of melatonin between sexes; and (4) most results were highly heterogeneous, but subgroup analysis was not done in this paper due to various limitations. The results of our sensitivity analysis (Figures 13–15) showed that none of the articles should be rejected because of the excessive influence on the pooled SMD, which confirmed stable results of Scr, BUN, and UAER.

## 5. Conclusions

Melatonin can protect renal function and delay pathological deterioration to achieve the aim of treating DM. Moreover, this study can provide preclinical reference for melatonin treatment of diabetic nephropathy. Considering the low quality and limited number of the included studies, more strictly designed studies are needed to examine the efficacy of melatonin as an antidiabetic nephropathy drug in the future.

## Figures and Tables

**Figure 3:**
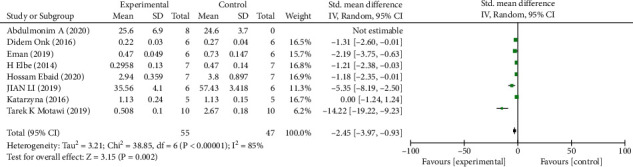
The meta-analysis results of melatonin for Scr [7, 20, 23–26, 28, 32, 33].

**Figure 4:**
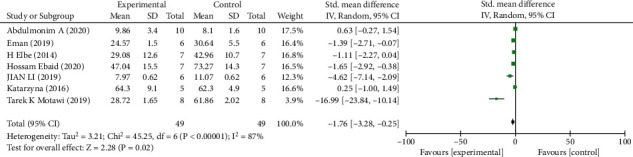
The meta-analysis results of melatonin for BUN [7, 20, 23, 26, 28, 32, 33].

**Figure 5:**
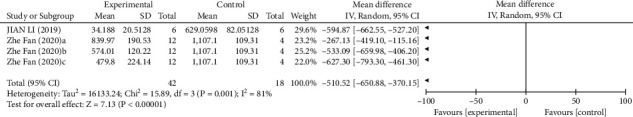
The meta-analysis results of melatonin for UAER [28, 31].

**Figure 6:**
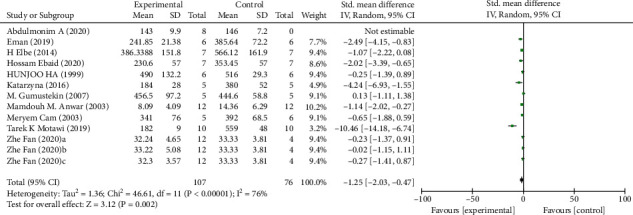
The meta-analysis results of melatonin for FBG [7, 14, 20, 23, 26, 27, 29–33].

**Figure 7:**
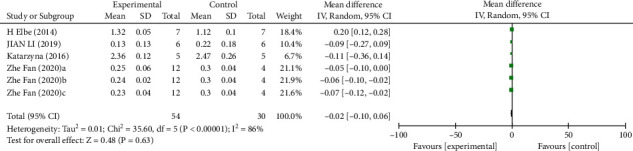
The meta-analysis results of melatonin for kidney weight [7, 20, 28, 31].

**Figure 8:**
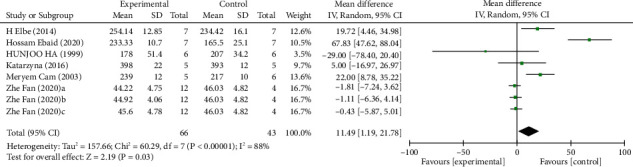
The meta-analysis results of melatonin for body weight [7, 20, 27, 30–32].

**Figure 9:**
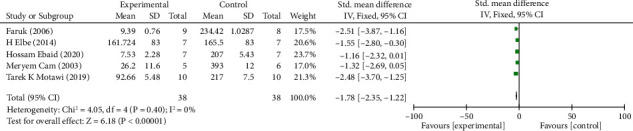
The meta-analysis results of melatonin for MDA [11, 20, 30, 32, 33].

**Figure 10:**
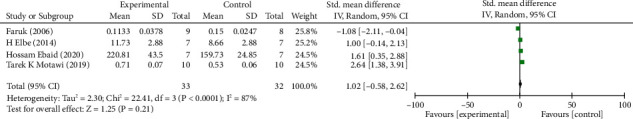
The meta-analysis results of melatonin for SOD [11, 20, 32, 33].

**Figure 11:**
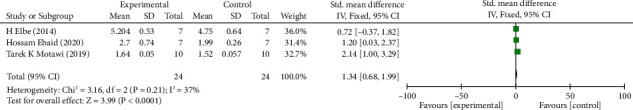
The meta-analysis results of melatonin for GSH [20, 32, 33].

**Figure 12:**
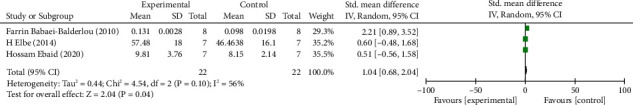
The meta-analysis results of melatonin for CAT [20, 32, 34].

**Table 1:** Characteristics of the included studies in the meta-analysis.

Study	Species	Sex	Weight (g)	Age (week)	*n* (T/no T)	Model (establish; modeling standard)	Treatment group (administration; dose; course of treatment)	Outcome index
Didem Onk (2016)^23^	SD rats	Male			6/6	SIJ STZ (50 mg/kg, BG > 400 mg/dL)	By intraperitoneal injection; 20 mg/kg/d; 1 week after establishing model (EM)	Scr
Eman (2019)^24^	Wistar albino rats	Male	100-150		6/6	SIJ STZ (50 mg/kg, diabetes was confirmed by glucotest strips)	po; 10 mg/kg/d; 2 weeks after EM	Scr, BUN, GLU
Faruk (2006)^9^	SD rats	Male	229.7 ± 32.9	11	9/8	SIJ STZ (35 mg/kg, BG ≥ 300 mg/dL)	By intraperitoneal injection; 10 mg/kg/d; 8 weeks after EM	MDA, SOD
Elbe (2014)^18^	Wistar albino rats	Male	300-350		7/7	SIJ STZ (45 mg/kg, BG ≥ 270 mg/dL)	By intraperitoneal injection; 10 mg/kg/d; 4 weeks and 2 days after EM	Scr, BUN, GLU, kidney weight, body weight, MDA, SOD, GSH, CAT
Ha (1999)^25^	SD rats	Male		7	6/6	Vein injection STZ (50 mg/kg, urine glucose concentrations > 2000 mg/dL)	po; melatonin supplement by 0.02% melatonin in drinking water; 4 weeks after EM	GLU, body weight
Li (2019)^26^	Wild-type (WT) mice	Male	250 ± 10	8	6/6	SIJ STZ (50 mg/kg, diabetes was confirmed by glucotest strips)	By intraperitoneal injection; 20 mg/kg/d; 12 weeks after EM	Scr, BUN, UAER, kidney weight
Katarzyna (2016)^5^	Obese diabetic ZDF rats (homozygous fa/fa)	Male		12	5/5	Obese diabetic ZDF rats (homozygous fa/fa)	po; received water treated with melatonin (20 mg/L) only during the 12-hour dark cycle to mimic physiological circadian changes in the hormone concentration in blood; 4 weeks after EM	Scr, BUN, GLU, kidney weight, body weight
Gumustekin (2007)^27^	Wistar rats	Male	170-300		5/5	SIJ STZ (45 mg/kg, BG > 300 mg/dL)	By intraperitoneal injection; 10 mg/kg; 5 days after EM	GLU
Anwar (2003)^12^	SD rats	Male	200-225		12/12	SIJ STZ (60 mg/kg, BG > 5 mmol/L)	By intraperitoneal injection; 200 *μ*g/kg/d; 2 weeks and 1 day after EM	GLU
Cam (2003)^28^	Wistar rats	Male	200-250		5/6	SIJ STZ (60 mg/kg, BG > 250 mg/dL)	By intraperitoneal injection; 200 *μ*g/kg/d; 4 weeks after EM	GLU, body weight, MDA
Fan (2020)a^29^	db/db mice	Male		9	12/12	db/db mice	iv; 200 mg/kg/d; 4 weeks after EM	UAER, GLU, kidney weight, body weight
Fan (2020)b^29^	db/db mice	Male		9	12/12	db/db mice	iv; 100 mg/kg/d; 4 weeks after EM	UAER, GLU, kidney weight, body weight
Fan (2020)c^29^	db/db mice	Male		9	12/12	db/db mice	iv; 50 mg/kg/d; 4 weeks after EM	UAER, GLU, kidney weight, body weight
Abdulmonim (2020)^21^	Albino rats	Male	200-250		8/8	Nicotinamide (230 mg/kg) was injected, followed by STZ (65 mg/kg) 15 min later, BG > 110 mg/dL	po; 0.3 mg/kg/d; 8 weeks after EM	Scr, BUN, GLU
Ebaid (2020)^30^	Wistar albino rats	Male	160-190		7/7	SIJ STZ (50 mg/kg, BG > 200 mg/dL)	10 mg/kg/d; 6 weeks after EM	Scr, BUN, GLU, body weight, MDA, SOD, GSH, CAT
Motawi (2019)^31^	Wistar albino rats	Male	150-200		10/10	SIJ STZ (50 mg/kg, BG 460-500 mg/dL)	By intraperitoneal injection; 200 *μ*g/kg/d for 3 days before diabetes induction and continuously administered for 2 months	Scr, BUN, GLU, MDA, SOD, GSH
Farrin (2010)^32^	Wistar rats	Male	180-220		8/8	SIJ STZ (50 mg/kg, BG > 220 mg/dL)	By intraperitoneal injection; 10 mg/kg/d; 2 weeks after EM	CAT

## Data Availability

No data were used to support this study.
